# Testing the Hypothesis of Fire Use for Ecosystem Management by Neanderthal and Upper Palaeolithic Modern Human Populations

**DOI:** 10.1371/journal.pone.0009157

**Published:** 2010-02-11

**Authors:** Anne-Laure Daniau, Francesco d'Errico, Maria Fernanda Sánchez Goñi

**Affiliations:** 1 EPHE, CNRS UMR5805, EPOC, Université Bordeaux 1, Talence, France; 2 CNRS UMR5199 PACEA, Université Bordeaux 1, Talence, France; 3 Institute for Human Evolution, University of the Witwatersrand, Johannesburg, South Africa; Stanford University, United States of America

## Abstract

**Background:**

It has been proposed that a greater control and more extensive use of fire was one of the behavioral innovations that emerged in Africa among early Modern Humans, favouring their spread throughout the world and determining their eventual evolutionary success. We would expect, if extensive fire use for ecosystem management were a component of the modern human technical and cognitive package, as suggested for Australia, to find major disturbances in the natural biomass burning variability associated with the colonisation of Europe by Modern Humans.

**Methodology/Principal Findings:**

Analyses of microcharcoal preserved in two deep-sea cores located off Iberia and France were used to reconstruct changes in biomass burning between 70 and 10 kyr cal BP. Results indicate that fire regime follows the Dansgaard-Oeschger climatic variability and its impacts on fuel load. No major disturbance in natural fire regime variability is observed at the time of the arrival of Modern Humans in Europe or during the remainder of the Upper Palaeolithic (40–10 kyr cal BP).

**Conclusions/Significance:**

Results indicate that either Neanderthals and Modern humans did not influence fire regime or that, if they did, their respective influence was comparable at a regional scale, and not as pronounced as that observed in the biomass burning history of Southeast Asia.

## Introduction

The earliest convincing evidence for human control of fire dates back to 500 kyr BP in Europe [1] and 790 kyr BP in the Near East [2]. Campfires recently found at Qesem Cave, Israel, support the idea that hominins possessed a controlled use of fire by 400–200 kyr BP [3]. This finding confirms previous views that fire became a widespread technology at the beginning of the Middle Palaeolithic [4]. Older possible evidence exists [5], but remains controversial [4], [6]. In Palaeolithic times fire may have been used for heating, cooking or roasting plant and animal food, illumination, and for protection from predators [3], [7].

Traditionally considered an innovation introduced to Europe by agriculturalists, slash and burn is a well known practice ([8] and references herein) used to clear and maintain open vegetation, fertilize soil, and facilitate cattle grazing [9]. Fire-use for ecosystem management is not restricted to agrarian and herding societies: regular burning of landscape is reported for Native Americans and aboriginal people of Australia (“fire-stick farming”) to create favourable habitats for the foraging and the hunting of small and big game [10], [11]. Williams [12] reports the selective use of fire by Native American tribes to drive big game (deer, elk, bison) into small unburned areas for easier hunting; to attract game which like to dine on young grass [10]; to drive rabbits into small areas; and to obtain salt from grasses. Plant management by fire is also reported for production of straight branches for basketry by Californian Indian Tribes [13]. Australian Aborigines skilfully use fire in different environments during the annual round of hunting and gathering. They have a well-developed knowledge of how to produce ignition and control fire-spread extent by burning the vegetation at the beginning and the end of the dry season when rain and storms are likely to extinguish fires [14], [15]. Landscape burning is undertaken for a variety of purposes including clearing thick vegetation to facilitate travel, signalling, controlling insects and vermin, hunting and waging war, ceremonial activities, driving game, regenerating senescent vegetation, smoking animals from burrows and asphyxiating bats in caves (see a synthesis in [11]). Fire-stick farming creates fine-grained landscape mosaics with a greater amount of food by increasing small-animal diversity and consequent hunting productivity [16]. Hill and Baird [17] report the use of fire by aboriginal Kuku–Yalanji groups of the Australian rainforest during the dry season to encourage the growth of *Cycas media* whose seeds constitute an important source of carbohydrates.

Mesolithic hunter-gatherers are suspected to have manipulated vegetation in northwest Europe [18] by deliberately setting fires to improve the sight-lines for missile-based hunting and/or change vegetation to attract game species [19]. Bos and Urz [20] also consider Early Mesolithic people responsible for woodland opening by deliberate burning of the vegetation. Although it is likely that these techniques were not invented by Mesolithic hunter-gatherers, but rather stemmed from earlier traditions, evidence supporting this hypothesis remains exceedingly scant.

It has been proposed [21], [22] that a greater control and more extensive use of fire is one of the modern behavioural innovations that emerged in Africa among Modern Humans, that favored their spread throughout the world, and that determined their eventual evolutionary success. We would expect, if extensive fire use for ecosystem management were a component of the modern human technical and cognitive package, as suggested by some authors [23]–[25], to find major disturbances in the natural biomass burning variability associated with and following the colonisation of Eurasia by Modern Humans.

Fire regime during this time span is poorly known due to the limited number of well dated and continuous charcoal records. A large increase in fire activity around 50 kyr cal BP, recorded in marine cores collected in the Pacific Ocean, has been interpreted as the result of burning produced by Modern Human populations colonizing Southeast Asia [26], [27]. Similarly, high incidence of biomass burning indicated by charcoal records in Australia between 60–45 ka has been attributed to the widespread use of fire for vegetation clearance during the initial colonisation of this continent [28], [29]. Dryness recorded in Australia after 45 ka has been interpreted as a feedback associated with changes in land-surface properties caused by human modification of the vegetation cover through fire [30], [31]. These interpretations, however, have faced repeated challenges in the last few years [32]–[36].

This paper addresses for the first time the question of whether Palaeolithic communities modified natural biomass burning variability during Marine Isotope Stage (MIS) 3 and 2 (59.4–27.8 kyr cal BP and 27.8–14.7 kyr cal BP) in Europe and in particular whether Modern Human populations colonising this large area and replacing Neanderthals at the so-called Middle to Upper Palaeolithic transition introduced fire as a tool for ecosystem management, possibly leading to an advantage over Neanderthals.

**Figure 1:**
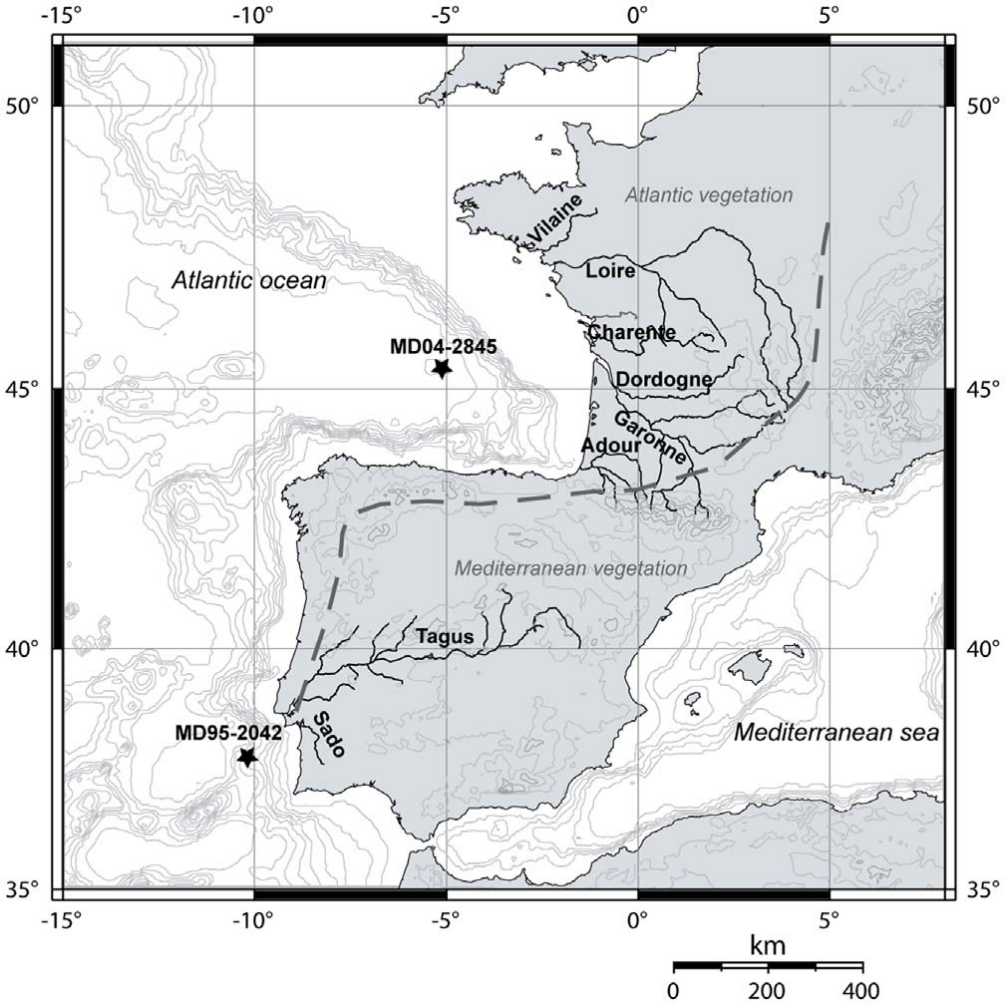
Location of cores MD04-2845 and MD95-2042 (filled stars) and main rivers draining Western France and Southwestern Iberia. Dashed line divides the Atlantic and Mediterranean biogeographical zones [118].

To explore this issue, analyses of microcharcoal preserved in two deep-sea cores located off Lisbon (MD95-2042) and Bordeaux (MD04-2845) (figure 1) were undertaken to assess changes in biomass burning in Southwestern Iberia and Western France, respectively (see Material and Methods section). Multiproxy studies of core MD95-2042 [37]–[43] and MD04-2845 [44] have documented the impact of the Dansgaard-Oeschger millennial-scale climatic variability and Heinrich Stadials (HSs) in the mid-latitudes of the eastern North Atlantic Ocean and Western Europe. Greenland Stadials (GSs) are characterised by a decrease in sea surface temperature (SST), high δ^18^O values of the planktonic foraminifera *Globigerina bulloides* and the development of the polar foraminifera *Neogloboquadrina pachyderma (s.)* left coiling. Greenland Interstadials (GIs) were characterised by warmer SST and low planktonic δ^18^O values. Extreme cooling episodes corresponding to HSs are marked by peaks of ice rafted debris (IRD), the strongest increase in planktonic δ^18^O values and peaks in magnetic susceptibility.

The age models of these two sequences are well constrained by numerous ^14^C AMS dates. These records provide a unique opportunity to correlate climatic fluctuations in the North East Atlantic region, vegetation change, palaeofire history, and human population dynamics during the MIS 3 and 2.

### Chronological and Cultural Framework

Neanderthal demise and the colonisation of Europe by Modern Humans are the object of intense debate in the palaeoanthropological and archeological literature. This debate principally concerns the taxonomic affiliation of the human populations responsible for the archaeological “cultures” dated to this period, the timing of this population replacement in each region of Europe, the possible role of climatic changes, and the nature of the biological and cultural interactions that led, in the end, to Neanderthal extinction. A large consensus exists around the notion that, in Europe, the Mousterian was solely made by Neanderthals [45], [46]. Many would also agree that the Chatelperronian, the only “transitional technocomplex” associated, at two sites, with diagnostic human remains was also made by Neanderthals [47], and that the Aurignacian should be attributed to Modern Humans [48], [49]. However, no human remains are securely dated to the first phases of the Aurignacian, now called Protoaurignacian [50] and relatively few to the subsequent, so called “Ancient Aurignacian”, which leaves open the possibility of a significant biological Neanderthal input on the first wave of modern colonisers [51]–[53]. In addition, some authors remain fervent partisans of an early colonisation of Europe by Moderns at ca. 39 kyr ^14^C BP and a subsequent acculturation of late Neanderthals [54], [55].

Creating a consensual chronological framework for this crucial population event is made difficult by the known limits of radiocarbon dating and calibration methods for the period before 26 kyr cal BP [56], [57] as well as by disagreement on the cultural attribution of key sites. Reappraisal of a number of these sites has challenged the existence of a diagnostic Aurignacian older than 36.5 kyr ^14^C BP (42 kyr cal BP) in Western Europe [58]–[60] and has shown that the Chatelperronian, interpreted in the past as resulting from acculturation of Neanderthals by Modern immigrants, is most probably older than the first Aurignacian. The more recent reliably dated Mousterian sites in France are not younger than 36 kyr ^14^C BP (41 kyr cal BP) and 36–34 kyr ^14^C BP (39–34 kyr cal BP) for the Chatelperronian [60], [61].

**Figure 2:**
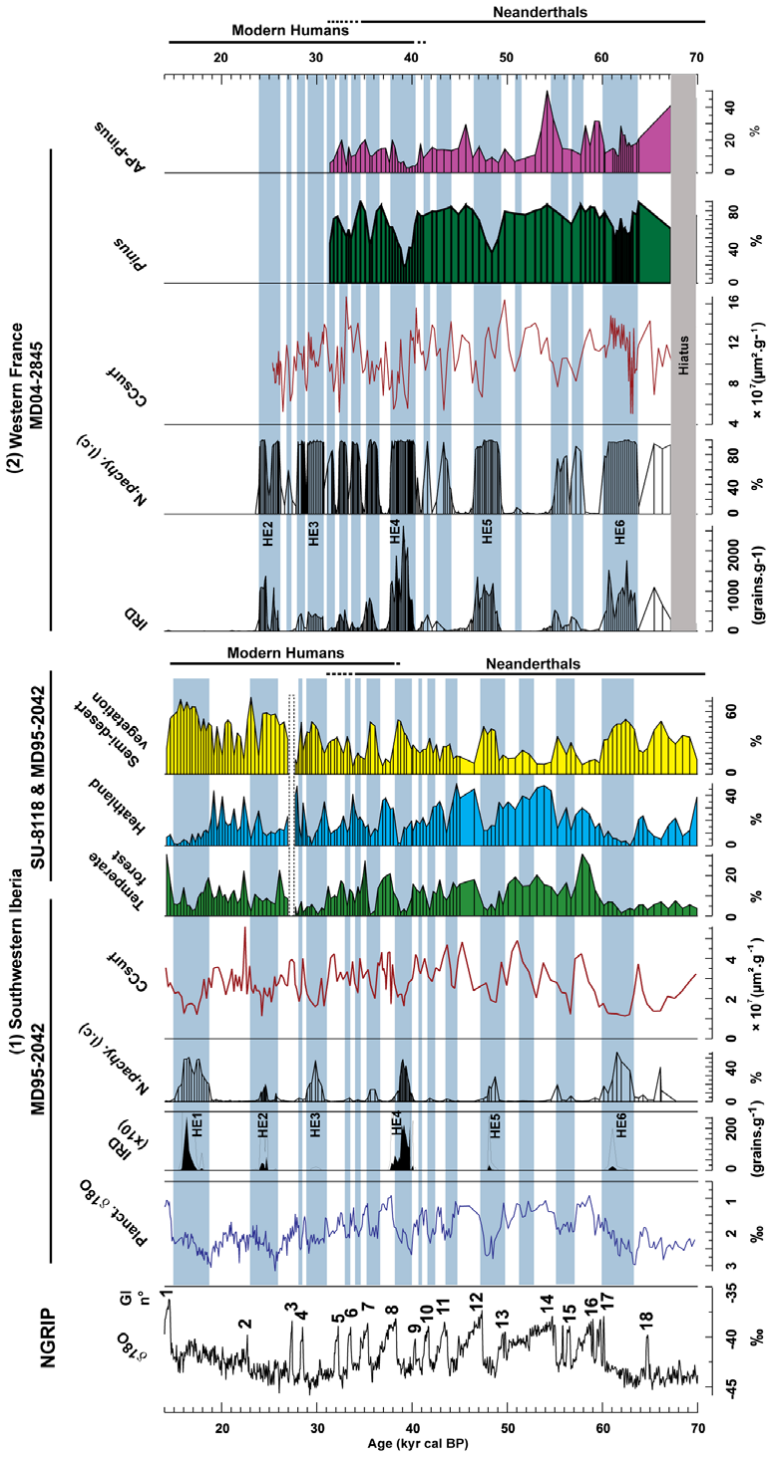
Comparison between concentrations of microcharcoal surface area (CCsurf) and climatic proxies of core MD95-2042 (Southwestern Iberia) (1) and MD04-2845 (Western France) (2). All records are plotted versus age. From left to right: (a) the NorthGRIP oxygen isotopic curve [119]; Southwestern Iberia: (b) the oxygen isotopic curve of the planktonic foraminifera *Globigerina bulloides* reflecting sea surface temperature and salinity changes [41], (c) the concentration curve of the ice rafted debris (IRD) and (d) the percentage curve of the polar foraminifera *Neogloboquadrina pachyderma (s)* left coiling[38], (e) the concentrations of microcharcoal surface area (CCsurf), (f) to (h) pollen percentage curve of: temperate forest including Mediterranean plants; Ericaceae (heather); semi-desert vegetation (*Artemisia*, Chenopodiaceae, Ephedra). The pollen data for core MD95-2042 are from [43]. The pollen data for the interval 14–25 kyr cal BP are from the twin core SU81-18 [120]. Western France: (a) the concentration curve of IRD, (b) the percentage curve of the polar foraminifera *N. pachyderma (s)* left coiling, (c) the concentrations of microcharcoal surface area (CCsurf), (d-e) the pollen percentage curve of *Pinus* and Arboreal Pollen (mainly composed of *Picea*, *Abies*, *Betula*, Cupressaceae, Hippophäe, deciduous *Quercus*, *Carpinus* and *Corylus*; *Pinus* excluded: *Pinus* pollen type is overrepresented in marine cores which precludes its inclusion in the calculation of AP percentages [44]. Grey band indicates a sedimentary hiatus in this core. The chronological extent of the Neanderthal and Modern Human populations are reported for the two regions. Blue bands indicate Heinrich Stadials (HSs) and other Greenland Stadials (GSs). HSs are identified on the basis of peaks in ice rafted debris (IRD), high percentages of the polar foraminifera (N. *pachyderma* (s.) and AMS ^14^C ages. GI numbers indicates Greenland Interstadials.

Climatically, the beginning of the Aurignacian (figure 2) in France has been related to the onset of the HS 4 (36.5 kyr ^14^C BP; ca 40 kyr cal BP), or the temperate phases (GIs 9–10) immediately preceding HS 4 [52], [61]. Radiocarbon dates indicate this culture persisted in Europe during the following temperate GIs 5–8 and cold/dry episodes preceding HS 3. Iberia represents, perhaps with other areas of Europe, a special case. The Aurignacian is attested in the North of the peninsula since at least 36.5 kyr ^14^C BP but absent in the South of Iberia before 33.5 kyr ^14^C BP (ca 38 kyr cal. BP), i.e. before HS 4 [58], [61] (figure 2). Although a few Mousterian sites seem to persist in Northern Iberia shortly after the emergence of the Aurignacian [62], [63] no archeologically detectable traces of this technology are found in the North after the HS 4. Neanderthals, in contrast, persist in the Southern Iberia at least until 30–32 kyr ^14^C BP [52], [61], [64], [65], which covers GI 8 and GI 5 event (figure 2). The gap between the North and the South is generally interpreted as evidence for a delayed colonisation of Southern Iberia by Modern Humans. The aridity and consequent low biomass produced by the HS 4 event on Central and Southern Iberia have been proposed as the main reasons for the late arrival of Modern Humans in the South [61], [66], [67]. For the sake of this study we accept the consensual view that Neanderthal demise occurred at ca. 34–33 kyr ^14^C BP in Western Europe and at ca. 30 kyr ^14^C BP in the South of Iberia.

## Results

**Figure 3:**
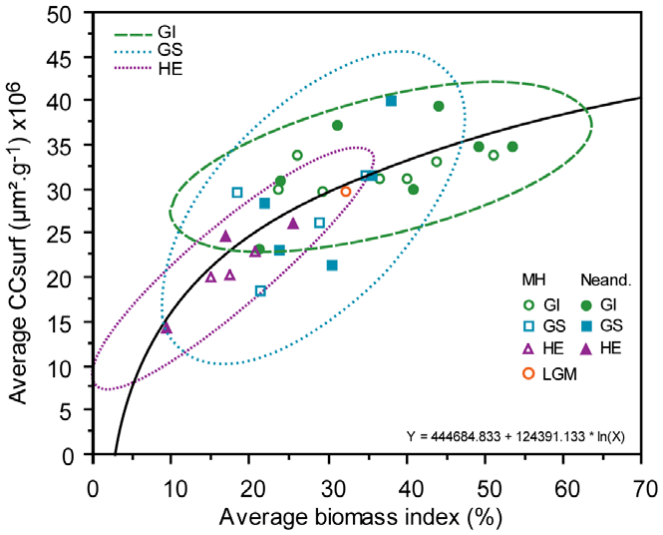
Average surface area of microcharcoal concentration (Average CCsurf) of core MD95-2042 *versus* average biomass index (Average biomass index) for each Greenland Interstadial (GI) and Stadial (GS) including Heinrich Stadials (HSs). LGM: Last Glacial Maximum. The biomass index for core MD95-2042 is determined by the sum of pollen percentages of Ericaceae and open Mediterranean forest. MH: Modern Humans, Neand: Neanderthals. The confidence ellipsoid at 95% is reported for each GI, GS and HS.

Concentrations of microcharcoal surface area (CCsurf) in core MD95-2042 (figure 2.1) reveal that the evolution of fire regime in Southwestern Iberia was in phase with vegetation shifts between GIs and GSs. High fire regime (see materials and methods for a definition) was contemporaneous with relatively warm and wet climatic phases (GIs), characterised by the development of an open Mediterranean forest and heathland. Low fire regime is observed during cold and dry climatic phases (GS including HS) characterised by semi-desert vegetation (*Artemisia*, Chenopodiaceae, *Ephedra*) [68]. As total plant biomass in forest and heathland communities is generally higher than open ground formations [69], these changes indicate the association of increased biomass accumulation with higher fire regime. CCsurf and biomass index, as reflected in the sum of arboreal Mediterranean taxa and heathland percentages, are strongly correlated (figure 3) and best fits a logarithmic function (*b* = 0.79, P<0.0001). This pattern indicates that in this region fuel availability, determined by climatically driven variations in vegetation biomass, is the main factor behind fire regime variation.

CCsurf from core MD04-2845 located off Bordeaux (figure 2.2) broadly follows the same pattern observed in the southern core for GSs and GIs. However, the correlation between warm/wet climatic conditions and corresponding fuel/fire regimes are not, after 40 kyr cal BP, as strongly expressed as in core MD95-2042. All HSs also reveal a more complex evolution, with a peak phase of CCsurf bracketed by two episodes of low fire regimes. Vegetation in Western France is mainly characterised by the expansions of coniferous forest (*Pinus, Abies, Picea*) and Atlantic forest (deciduous *Quercus*, *Betula*) during GIs and steppic vegetation during GSs [44]. The correlation of CCsurf and Arboreal Pollen (*b* = 0.525, P<0.0174, excluding *Pinus*) suggests that the fire regime of the last glacial period was still determined by changes in biomass. *Pinus* woodland certainly contributed to the biomass burned but the overrepresentation of this pollen taxon in marine cores precludes effective correlation tests without pre-corrections. Note that some GIs are characterised by relatively weak biomass/low fire regime and some HSs by relatively strong biomass/high fire regime. This is the case with HS 6, during which the expansion of *Picea* and *Pinus* woodlands may reflect the establishment of a fire-prone ecosystem such as that of boreal forest [70]. The three-phase structure that characterises HS suggests a more complex relationship in Western France between factors conditioning fire regimes (biomass, vegetation composition, precipitation, temperature, and lightning occurrence) [70]. The first decrease in fire regimes within HS may be due to a concomitant reduction in fuel and an increase in humidity, whereas the subsequent peak could reflect an increase in drought conditions and/or lightning associated with increasing fuel. The final reduction in fire regime could be the result of a new relative increase in humidity.

**Figure 4:**
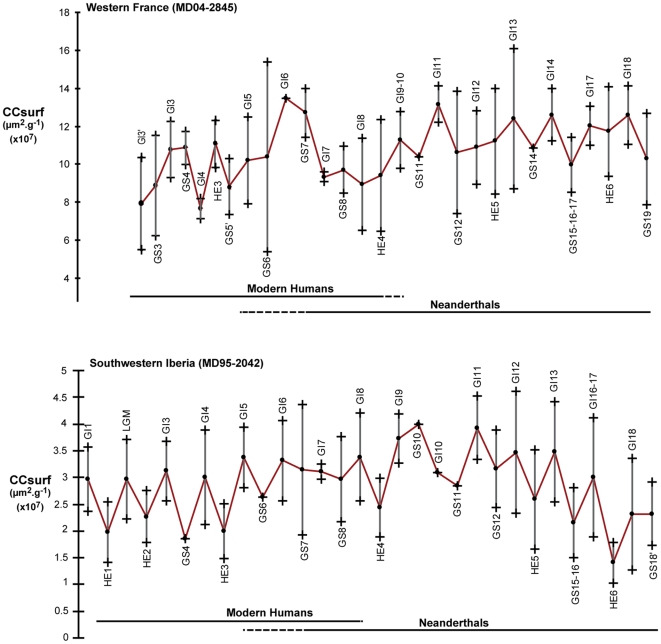
Evolution of average surface area of microcharcoal (CCsurf) with standard deviation reported for each Greenland Stadial (GS), including Heinrich Stadials (HSs), and Greenland Interstadial (GI) in core MD04-2845 and MD95-2042 compared to the chronological extent of Neanderthals and Modern humans.

The described pattern persists after 41–42 kyr cal BP (GIs 9–10) in core MD04-2845, and after 38 kyr cal BP (GI 8) in core MD95-2042, the time accepted by most authors for the arrival of Modern Humans in France and Southern Iberia, respectively (figure 2). The only possible exception is represented by CCsurf signal for the beginning of GS 7 in the French core, which appears anomalously high for a GS. This, however, happens 6,000 years after the arrival of Modern Humans in Western France and is not recorded in the Southwestern Iberian margin. Both Neanderthals and Modern Humans are associated in Europe with periods of relatively high biomass and fire regime and periods of weak biomass and low fire regime (Figure 3). This implies that the observed large scale variations in fire regime were not determined by the population replacement. Figure 4 reveals a long term decreasing trend of fire regime in Western France between 70 and 25 kyr cal BP and, in Southwestern Iberia, an increasing fire regime until GI 10 followed by a long term decrease until 14 kyr cal BP. These long term trends also do not match the timing for the Neanderthal/Modern Human replacement.

## Discussion

Understanding cause-and-effect relationships between human-landscape interaction and regional fire regimes in Southeast Asia and Australia remains elusive despite both the abundant charcoal records available and the relatively simple settlement histories of these regions compared to Europe.

The strong increase in microcharcoal concentration observed in marine cores from Southeast Asia has been used to argue in favour of an extensive use of fire at around 50 kyr cal BP by modern human colonisers to clear vegetation and facilitate hunting [26], [27]. This would have perturbed natural fire regimes and dramatically changed vegetation composition [71]. Pollen analysis of the archaeological sequence from Niah cave (Malaysia), dated to the MIS 3, reveals an increase in *Justicia*, a fire-favoured taxon, associated with forest rich phases [72]. This has been interpreted as reflecting management of forest by modern human settlers to stimulate the growth of tubers and edible plants, and hunt animals attracted to clearings [73]. A similar interpretation has been suggested for the increase in biomass burning indicated by charcoal records in Australia between 60–45 ka and the following dryness observed in the area [30], [31]. Several lines of evidence, however, have recently challenged the anthropogenic hypothesis: 1) major changes in fire regimes have been documented by charcoal records for periods prior to the arrival of modern humans [32], [34]; 2) changes in the ENSO variability and its impact on rainfall may explain the increase in natural fire [33]; 3) a broadly-synchronous transition to more xerophytic vegetation is observed in New Caledonia, a region not colonised until 3,000 years ago [35]; 4) climate-model experiments show that feedbacks associated with fires, even catastrophic fires, would have been insufficient to dry out the region [36].

Contrary to Australia, where colonisers penetrated an unpopulated territory, Modern Humans entering Europe met a human population, the Neanderthals, well adapted to mid-latitude environments. Neanderthal knowledge of pyrotechnology is fully compatible with the hypothesis of their use of fire for ecosystem management. Numerous hearths have been found at Mousterian sites and in a number of cases there is clear evidence that they were deliberately constructed, maintained, and re-used [74]–[77]. Neanderthals also used fire to harden wooden spears [78] and to process birch pitch to haft stone tools [79], [80].

Fire use for ecosystem management could be 1) a subsistence strategy already in possession of Neanderthals when Moderns arrived in Europe, 2) an innovation introduced in this region by moderns, 3) developed by them after their arrival, or 4) absent from both technical packages either because not a part of their cultural heritage or because ineffective in the European latitudes during MIS 3-2.

Our results show that contrary to Southeast Asia, no major increase in fire regime is recorded in Southwestern Iberia or in Western France at the onset or after the colonisation of these regions by Modern Human populations. CCsurf values associated in Southeast Asia with Modern Human impact are twice as great as the highest figures recorded in the same sequences for the period before colonisation by Modern Humans. Such a dramatic increase is not observed in our records. Also, no shift is observed in the vegetation apart from that expected by the impact of the millennial scale climatic variability on plant communities, and no increase in taxa that might be related to an increase in fire. Although the Southeast Asian and the European trends are difficult to compare considering the different latitudinal, paleoclimatic and vegetation settings, the coincidence in the former area between the peopling event and the increase in biomass burning makes it conceivable that the two phenomena are related in some way.

Our results strongly argue against the view that Neanderthals and Modern Humans were the driving factor of the large scale variations in fire regime observed in our records, which were clearly governed by the D-O millennial-scale climatic variability and its impact on fuel load. However, we cannot rule out at this stage the possibility that either one or both populations used fire for ecosystem management in ways that did not significantly affect the natural fire trend.

A number of quantitative analyses of the effects of Aboriginal fire treatment in Australia (e.g. [15], [16], [81]) have shown that under an anthropogenic regime the frequency of fire increases, but the intensity and extent of burns declines dramatically, resulting in a decrease in biomass burning ([15], [81], [82]. Management of fire by Aboriginal people leads to fragmentation of the landscape, reducing fuel and therefore reducing fire. In contrast, the cessation of traditional aboriginal burning practices enhances vegetation development and fuel accumulation, allowing the occurrence of large and intense fires.

If this were the case in Europe and our record could detect such a change we should find a decrease in the biomass/CCsurf ratio after the introduction of this ecosystem management practice. The question, however, is whether such a decrease may be detected by regional sequences. Marlon et al. [83] have shown that the decrease in biomass burning, produced by expansion of intensive grazing, agriculture and fire management over the past 150 years, is recorded at a regional and global scale, but in this case, of course, we are dealing with a massive impact of humans on a variety of ecosystems.

Considering the relatively low population density predicted for Palaeolithic Europe [84], anthropogenic ignition sources may have simply amplified or otherwise subtly altered natural rhythms of fire frequency and intensity. If this were the case, a shift to an anthropogenic fire regime could only be detected by comparing the regional fire signal given by marine cores with multiple well dated continental sequences recording local fire frequency, intensity and resulting landscape patchiness.

### Conclusion

Extensive use of fire for ecosystem management was probably a component of the technical package of Modern Humans during their colonisation of Southeast Asia. Our study shows that fire regimes in Western Europe between 70 ka and 10 ka were mainly driven by the D-O millennial-scale climatic variability and its impacts on fuel load. At a macro level at least, the colonisation of Western Europe by Anatomically Modern Humans did not have a detectable impact on fire regimes. This, however, does not mean that Neanderthals and/or Modern Humans did not use fire for ecosystem management but rather that, if this were indeed the case, the impact on the environment of fire use is not detectable in our records, and was certainly not as pronounced as it was in the biomass burning history of Southeast Asia.

## Materials and Methods

### Core Location, Sampling and Chronostratigraphy

Deep-sea core MD95-2042 (37°14′50N, 10°11′00W; 3146 m water depth) was retrieved off Lisbon, 140 km from the nearest coast line on a nearly flat continental rise (figure 1). The sediments are mainly composed of clayey mud, with 20–40% carbonate content and <1% organic matter [37], [39]. Deep-sea core MD04-2845 (45°20′86N; 5°13′17W, 4175 m water depth) was retrieved 350 km west of Bordeaux from the Gascogne seamount (Figure 1). Sediments are mainly composed of clayey mud with sparse silty laminations, with 10–65% carbonate content and <1% organic carbon [70]. These two cores have shown a well preserved hemipelagic sedimentary sequence not perturbed by turbidites [70], [85].

For the purpose of this study we focus on the time span between 70 and 10 kyr cal BP, which covers the last 30,000 years of Neanderthal history and the whole Pleistocene occupation of Europe by Modern humans. The age model of core MD95-2042 covering this period is based on 10 age control points [41], [86], [87], 16 AMS ^14^C ages [56], and follows GISP2 and GRIPSS09 sea ice core chronology. The age model for core MD04-2845 is based on 10 AMS ^14^C ages and correlation of the onset of GIs and the boundaries of HSs with those identified and dated in core MD95-2042 [44]. Core MD95-2042 was sampled for microcharcoal analysis every 10 cm between 420 cm down to 2000 cm, and every 5 cm between 1300 and 1419 cm [68] giving a mean resolution of <400 years (40–1200 years). Core MD04-2845 was sampled for microcharcoal analysis every 5 cm between 1740 cm (MIS 4) and 760 cm (beginning of HS 2) giving a mean resolution of 500 years [70].

### Microcharcoal Origin, Deposition and Preservation in Deep-Sea Core

At present, fires in Portugal and Spain occur during the dry summer season (June to August) ([88], [89], http://www.incendiosforestales.org/estadisticas.htm], in particular during extreme synoptic situations characterised by hot and dry south-easterly winds on the Iberian Peninsula [90]. Fires in France are best represented in the southeast Mediterranean region during the summer season. Western France is generally not affected by large fires, but fire lighting during storms exists [http://www.feudeforet.org/].

To trace fire regime variability, we analyse microcharcoal preserved in these two deep-sea cores. The term “microcharcoal” refers to small carbonised particles produced during vegetation fires [91] and transported by aeolian and fluvial agents from the combustion site to the sedimentation basin. In lake, peat bog or ocean sedimentary contexts, charcoal preserves well due to its relatively high resistance to chemical [92]–[94] and microbial decomposition [95], [96]. Microcharcoal sedimentation in marine environments can be compared to that of pollen, which is deposited in a matter of weeks on the ocean floor as a part of the marine snow [97], [98]. Works conducted by a number of scholars (see [99],[100] for wind transport and [101]–[103] for water transport) have shown that aeolian and fluvial transport occur at most in a matter of months or few years. This implies that no significant time lag exists, at a resolution of centuries or millennia, between production and deposition of microcharcoal [68].

Rivers are at present the main sources of fine sediments (including microcharcoal and pollen) to the Bay of Biscay and the Iberian margin [104], [105]. This was certainly the case during the last glacial periods because the North Atlantic westerlies were the dominant winds over Western Europe. This precludes a significant input of airborne microcharcoal in both cores [68], [70]. Microcharcoal preserved in deep-sea core MD95-2042 was most likely recruited by the Tagus and the Sado rivers from the fires occurring in their hydrographic basins. Microcharcoal of deep-sea core MD04-2845 was recruited by the rivers draining the close continent: Gironde, Garonne, Dordogne, Loire, Vilaine, Charente and Adour (figure 1).

### Microcharcoal Analysis

The microcharcoal extraction technique consists of a chemical treatment of 5 mL 37% HCl, 5 mL 68% HNO_3_ and 10 mL of 33% H_2_O_2_ performed over 24 h on about 0.2 g of dried sediment, followed by a dilution of 0.1 applied to the residue. The suspension is then filtered onto a membrane of 0.45 µm porosity and 47 mm in diameter. A portion of this membrane is mounted onto a slide.

Identification of microcharcoal is performed using an automated image analysis in transmitted light and following the criteria proposed by [106] who identifies charcoal as being black, opaque, and angular with sharp edges. Since erosion of organic enriched sediment (including coal) from sedimentary basins can be a source of non-burnt and oxidised particles, called vitrinite [107], which can appear black in transmitted light and be misidentified as burnt particles, petrographic analysis was conducted on randomly selected samples. Identification of unburned particles, characterised by the absence of plant structures and distinct level of reflectance, was used to set the bestfit threshold level to secure identification of microcharcoal by image analysis (for more detailed description of the method see [68] and [70]).

The concentration of microcharcoal surface area (CCsurf) is then calculated [26]. CCsurf represents the total surface area of microcharcoal per gram (µm^2^.g-1) and is given by the following equation: CCsurf  =  (P*Sp*Sr)/(D*W*Ss) where P is the number of pixels identified as charcoal by image analysis, Sp is the surface of a pixel, Sr is the area of the filter, D is the factor of dilution, W is the weight of dry sediment, Ss is the area scanned by the microscope. Using CCsurf instead of the number of microcharcoal per gram prevents overrepresentation due to taphonomic processes [108], [109].

The fire regime of a given environment is defined by the variables that influence species survival: fuel consumption and fire spread patterns, which determine the observed fire type (surface, ground, crown fires or a mixture of the three) and size; fire intensity (i.e. the energy release); severity (i.e. the impact on ecosystem, for ex. the measure of tree mortality); frequency (i.e. the occurrence of fire in a given period and area); and fire season (determined by combined ignitions and low fuel moisture conditions) [110]. Fire frequency can be reconstructed in certain cases (see [111]), but most palaeorecords only provide an indication of relative changes in biomass burning. These changes are unlikely to have appeared without changes in one of the parameters controlling the fire regime. Therefore we can reasonably expect that, at regional and decadal to millennial scale [112], changes of biomass burning in our marine microcharcoal records reflect changes in fire regime.

CCsurf is commonly considered a reliable proxy for biomass burning [113]–[117]. However, variations of CCsurf in our cores could also reflect changes in microcharcoal input linked to changes of fluvial sedimentary input or dilution processes due to increase in terrigenous material. To verify that CCsurf was not affected by these processes and reflect fire regime variability, we calculated the sedimentation rate of both cores and compared it with CCsurf (Figure S1). This comparison reveals no common trends. We also correlated sedimentation rate with the mean charcoal surface for each climatic phase (Figure S2). If CCsurf were determined by changes in the sedimentary input or by charcoal dilution we should observe a strong positive or negative correlation between these variables, respectively. This is not the case. Finally, the decrease in CCsurf observed during GSs, including HSs, in both cores cannot be explained by a dilution created by IRD since a similar decrease is systematically observed during GSs in core MD95-2042 where IRD are absent. CCsurf variations appear therefore independent of sedimentation processes and represent a reliable proxy to describe changes of the fire regime of Southwestern Iberia and Western France for the period concerned (70–10 kyrs ago).

## Supporting Information

Figure S1 Variations of sedimentation rate and microcharcoal surface area concentrations (CCsurf) recorded in cores MD95-2042 (a) and MD04-2845 (b).
(9.53 MB TIF)

Figure S2 Correlation between the average concentration of microcharcoal surface area (Average CCsurf) and the sedimentation rates for climatic events identified between 70 and 14 kyr cal BP in cores MD95-2042 (a) and MD04-2845 (b).
(6.02 MB TIF)
