# Supplementary figures and images for: Testing the Hypothesis of Fire Use for Ecosystem Management by Neanderthal and Upper Palaeolithic Modern Human Populations
Source: PLoS One. 2010 Feb 11;5(2):e9157. doi: 10.1371/journal.pone.0009157 (PMC2820084; doi:10.1371/journal.pone.0009157)

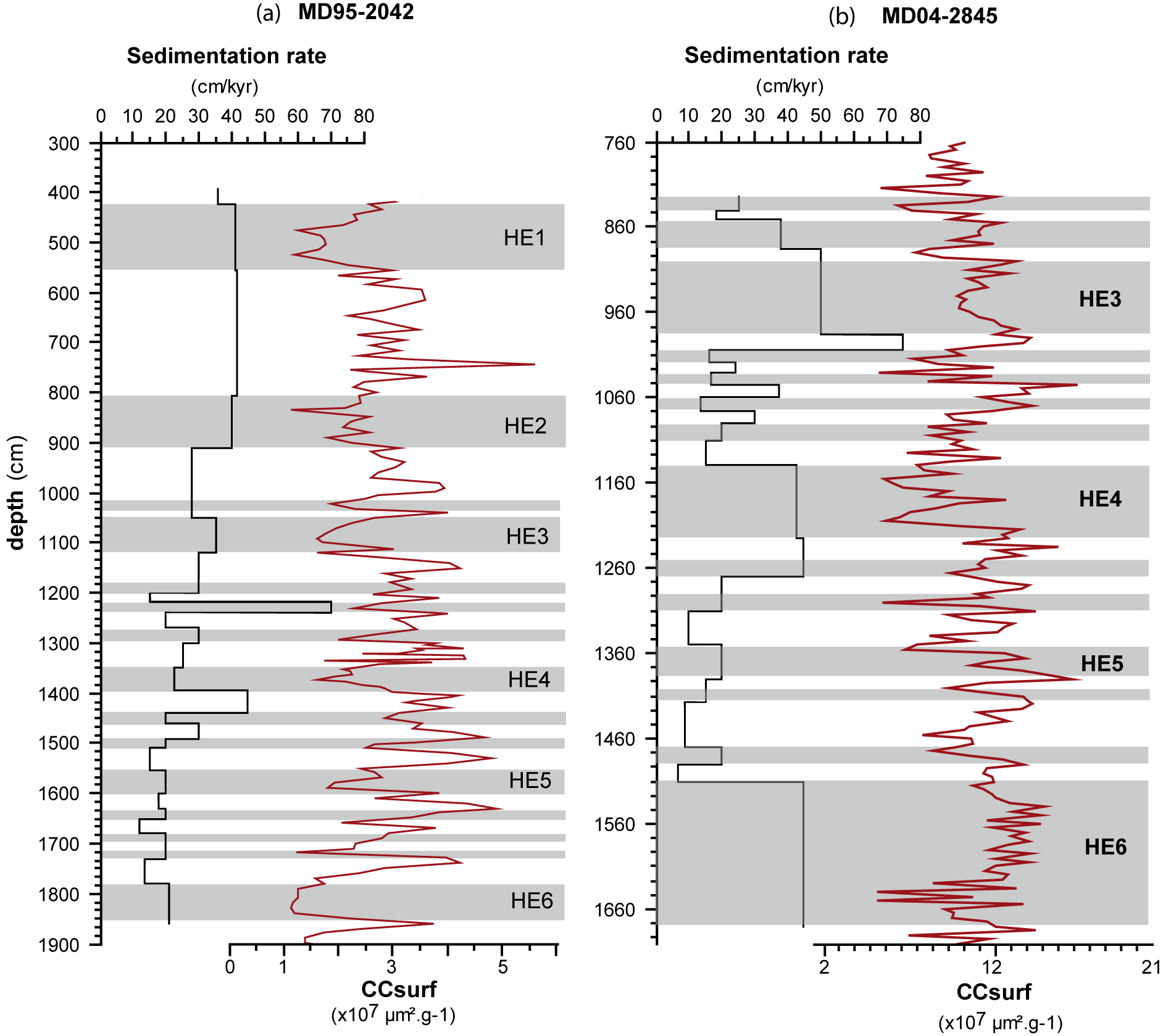

Supplement: Figure S1 — Variations of sedimentation rate and microcharcoal surface area concentrations (CCsurf) recorded in cores MD95-2042 (a) and MD04-2845 (b). (9.53 MB TIF) [file pone.0009157.s001.tif]

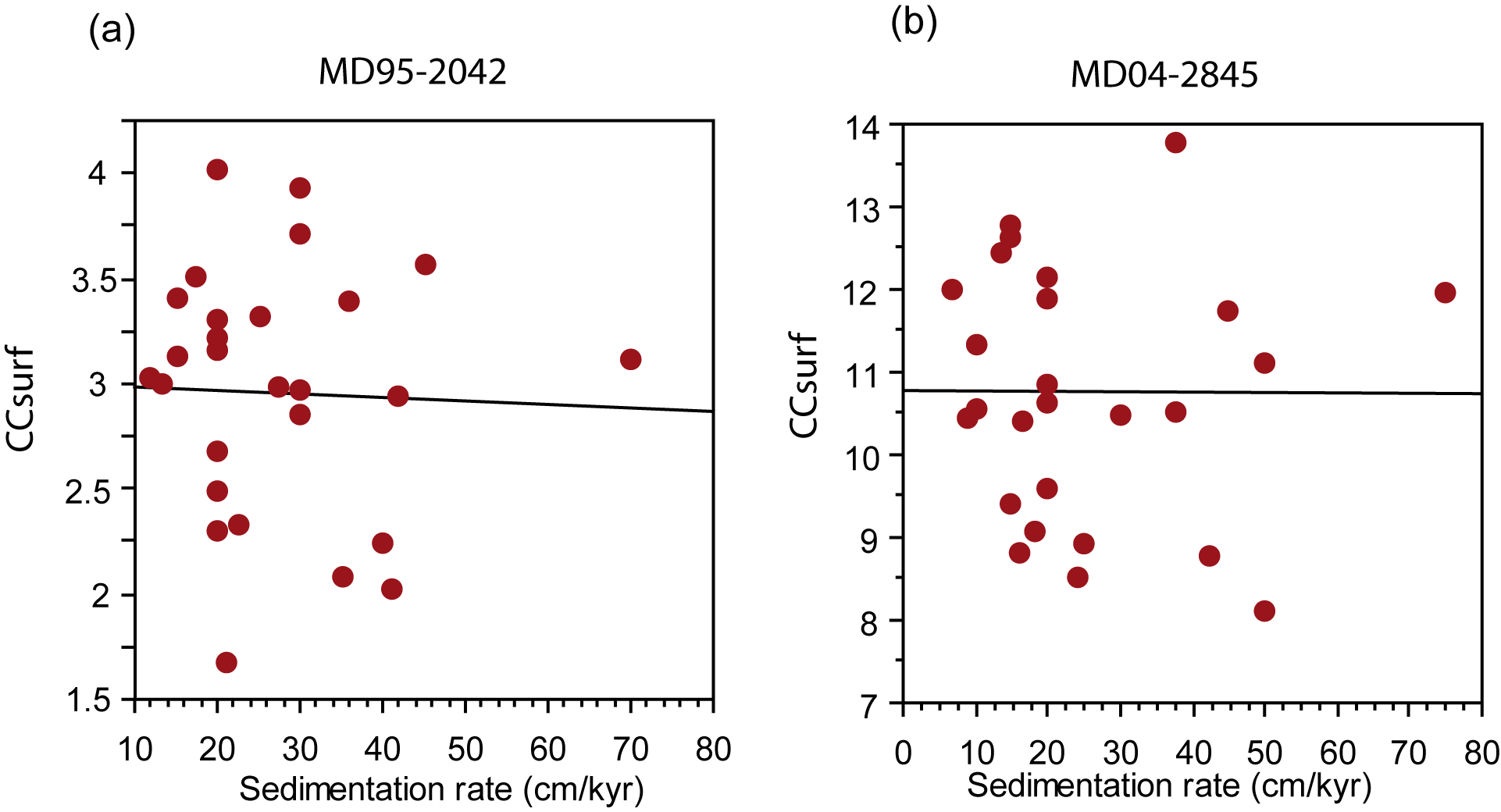

Supplement: Figure S2 — Correlation between the average concentration of microcharcoal surface area (Average CCsurf) and the sedimentation rates for climatic events identified between 70 and 14 kyr cal BP in cores MD95-2042 (a) and MD04-2845 (b). (6.02 MB TIF) [file pone.0009157.s002.tif]
